# Disruption of interferon-β production by the N^pro^ of atypical porcine pestivirus

**DOI:** 10.1080/21505594.2021.1880773

**Published:** 2021-02-04

**Authors:** Chunxiao Mou, Shuonan Pan, Huiguang Wu, Zhenhai Chen

**Affiliations:** a College of Veterinary Medicine, Yangzhou University, Yangzhou, Jiangsu Province, People’s Republic of China; b Joint International Research Laboratory of Agriculture and Agri-Product Safety, the Ministry of Education of China, Yangzhou University, Yangzhou, Jiangsu Province, People’s Republic of China; c Jiangsu Co-Innovation Center for Prevention and Control of Important Animal Infectious Diseases and Zoonoses, Yangzhou University, Yangzhou, Jiangsu Province, People’s Republic of China

**Keywords:** Atypical porcine pestivirus, nonstructural protein N^pro^, IFN-β production, IFN regulatory factor 3

## Abstract

Atypical porcine pestivirus (APPV) is an emerging porcine virus that threatens global swine production. Pestiviruses can prevent interferon (IFN) production to avoid the host innate immune response, and the N^pro^ viral protein can play a critical role. Knowledge of the host immune response to APPV infection is limited. Here, we showed that the IFN-β production was suppressed by APPV-N^pro^ and the IFN regulatory factor 3 (IRF3) promoter activity stimulated by adaptor molecules of the IFN-β signaling pathway was also inhibited in the APPV-N^pro^-expressed cells. The APPV-N^pro^ was able to interact with IRF3 and interfere the phosphorylation of IRF3, indicated that the IFN-β antagonism of APPV-N^pro^ mainly depended on blocking IRF3 activity. To identify the functional region of APPV-N^pro^, a panel of truncated APPV-N^pro^ was constructed, and its influence on the IRF3 activation was investigated. The results showed that the N-terminal 31–51 amino acids of APPV-N^pro^ were mainly associated with inhibition of the IFN-β response. Taken together, this is the first study focusing on elucidating the function of APPV protein by revealing a novel mechanism of N^pro^ in disruption of host IFN-β production, which will enlighten future study in addressing APPV pathogenesis and immune evasion.

## Introduction

Atypical porcine pestivirus (APPV) is a member of the *Flavivirus* family, which are positive-sense RNA viruses [1]. It is a highly variable enveloped virus containing a positive sense, single-stranded genome of about 11 to 12 kb in size. The genomic RNA contains 5ʹ and 3ʹ untranslated regions (UTRs), which are related to the replication and translation of RNA. The viral proteins were encoded in a large open reading frame (ORF), which is processed co- and post-translationally to produce four structural proteins (C, E^rns^, E1, and E2), as well as eight nonstructural proteins (N^pro^, p7, NS2A, NS2B, NS3, NS4A, NS4B, and NS5) [2]. APPV was initially identified as a novel and divergent porcine pestivirus in the United States, and then found in Europe and Asia, which is threatening the global pig production industry [3]. APPV in piglets with clinical signs of congenital tremor (CT) type A-II, which causes piglet growth retardation, starvation, and preweaning mortality [4]. Due to the limited knowledge of APPV, a better understanding of virus pathogenesis and immunology would benefit for controlling the virus infection.

Interferon (IFN) is essential for host defense against pathogens [5,6]. It is capable of controlling most, if not all, virus infections in the absence of adaptive immunity. During viral infection, the host pattern recognition receptors (PRRs) recognize the invading viruses and initiate an innate immune signaling cascades that results in the activation of transcription regulators, including IFN regulatory factors (IRFs) and nuclear factor κB (NF-κB). Several PRRs are able to recognize viral RNA such as Toll-like receptors and retinoic acid-inducible gene I-like receptors (RIG-I and MDA5) [7,8]. After viral RNA is recognized by RIG-I and MDA5, activated RIG-I and MDA5 are able to interact with mitochondrial antiviral signaling protein (MAVS) through 2CARD interaction. This interaction further induces the recruitment of downstream signaling molecules, including the TANK-binding kinase 1 (TBK1) [9,10]. In the TLR3 signaling pathway, the engagement of TLR3 leads to the oligomerization of the adaptor TICAM-1 (TRIF), which can activate the IRF3 and NF-κB [11]. The activated transcription factors are translocalized into nuclei, and then induce the interferons (IFNs) and other cytokines expression to regulate innate and adaptive antiviral immune responses.

Although hosts have highly efficient strategies to resist the virus invasion, viruses have also developed a variety of strategies to avoid or subvert the host defenses [12]. The pestiviruses can establish a persistent infection and enhance other viruses’ replication by suppressing IFN-I induction [13–16]. APPV is commonly found together with other viruses in persistently infected animals, which may be helpful for inducing those secondary infections [17,18]. In the polyprotein of pestiviruses, the first protein (N^pro^) is unique, which is an autoprotease and can cleave itself from the nascent polypeptide at the N^pro^/C site [19]. The ability of pestiviruses to subvert innate immune defenses was attributed to the viral N^pro^ protein that prevents IFN-I induction [13–16]. The N^pro^ of the classical swine fever virus (CSFV) and bovine viral diarrhea virus (BVDV) are involved in the down-regulation of IFN-β responses [20]. Therefore, we suspected that APPV-N^pro^ is also involved in regulating the innate immune responses in APPV infection.

In this study, the association between APPV-N^pro^ and IFN-β antiviral responses was investigated. We analyzed the disruption of IFN-β production by APPV-N^pro^ and detected the interaction between APPV-N^pro^ and the key sensor of IFN-β signaling pathway. Further, the functional region of APPV-N^pro^ inhibiting the IFN response was identified. These results revealed a novel mechanism of APPV-N^pro^ antagonizing the innate immune responses.

## Materials and methods

### Cells and plasmids

The swine testicular (ST) cells and human embryonic kidney (HEK293T) cells were cultured in the Dulbecco’s minimal essential medium (DMEM) (Thermo Fisher Scientific, China) with 10% fetal bovine serum (Hyclone, USA). The ST-EGFP-APPV-N^pro^ cell line was cultured in the medium supplemented with 300 μg/ml Hygromycin B (Thermo Fisher Scientific, China). All cells were incubated in the presence of 5% CO_2_ at 37°C.

APPV-N^pro^ gene was amplified from the cDNA of the APPV and the CSFV-N^pro^ gene was amplified from the cDNA of CSFV. The PCR fragments were inserted into the pCAGGS-Flag or pCAGGS-HA vector. Truncated APPV-N^pro^ genes (1–60, 61–120, 121–180, 21–180, 31–180, 52–180, and 61–180 nt) were cloned into the pCAGGS-Flag vector as shown in. A series of human innate immune genes encoding TRIF, RIG-I, MAVS, IRF3, and TBK1 were amplified from HEK293T cells and cloned into pCAGGS-Flag or pCAGGS-HA vector. Similarly, the porcine innate immune genes encoding TRIF, RIG-I, MAVS, IRF3, and TBK1 were amplified from ST cells and cloned into pCAGGS-Flag vector. Constructs were confirmed by DNA sequencing.

### Construction of ST-EGFP-APPV-N^pro^ cell line

The construction of recombinant plasmid pLVX-EGFP-APPV-N^pro^ was shown in. To generate lentiviruses expressing APPV-N^pro^, HEK293T cells were co-transfected with pLVX-EGFP-APPV-N^pro^, packaging vector psPAX2, and envelop vector pMD2.G by Lipofectamine 3000 (Thermo Fisher Scientific, China). Culture supernatants containing lentiviruses were harvested at 24 h and 48 h post-transfection, passed through a 0.45 μm-pore-size filter. ST cells were infected by lentiviruses expressing EGFP-APPV-N^pro^, and then subjected to selection with Hygromycin B to generate ST-EGFP-APPV-N^pro^ stable cell line. Furthermore, the fluorescence in ST-EGFP-APPV-N^pro^ cells and ST cells were observed using fluorescence microscope. The expression of fusion protein in the ST-EGFP-APPV-N^pro^ cells was analyzed by western blot.

### Luciferase assay

The luciferase assay for detecting IFN-β, NF-κB and IRF3 promoter activities were carried out as described before [21]. The plasmids pIFN-β-luc, pIRF3-luc, pNF-κB-luc, and pRL-TK were purchased from Promega. Briefly, the viral protein expression plasmids together with luciferase plasmids and pRL-TK were co-transfected into HEK293T cells. The pRL-TK vector provided constitutive expression of Renilla luciferase. After 24 h post-transfection, cells were mock- or treated with 200 ng Poly (I:C) (Sigma, China) for 12 h. The luciferase activity in cells lysates were detected using the Dual-Luciferase assay system (Promega, China) according to the manufacturer’s instructions.

### Co-immunoprecipitation

HEK293T cells were co-transfected with pCAGGS-HA-IRF3, pCAGGS-Flag-APPV-N^pro^, or truncated APPV-N^pro^ expression plasmids. The ST-EGFP-APPV-N^pro^ cells were transfected with pCAGGS-Flag-Pig-IRF3. After 24 h post-transfection, the cells were lysed with lysis buffer (20 mM Tris-HCl, 300 mM NaCl, 2.5 mM MgCl_2_, 10% glycerol, 0.1% Nonidet P40, protease inhibitor cocktail, and phosphatase inhibitor (Sigma)). The lysate was centrifuged and incubated with 2 μL anti-Flag M2 magnetic beads or anti-HA magnetic beads (Sigma) for 4 h at 4°C on a roller. The magnetic beads were washed three times with lysis buffer, and then the immunoprecipitates were harvested and analyzed by western blot.

### Western blot

Cells were lysed through cell lysis buffer with phenylmethylsulfonyl fluoride (PMSF; Beyotime, China). The samples were performed by SDS-PAGE and western blot. Briefly, the cell lysates were resolved in 12% polyacrylamide gel, and then transferred to PVDF membranes (Millipore, China). After blocked in Tris-buffered saline containing 0.1% Tween-20 (TBST) and 10% nonfat milk for 2 h, the membranes were incubated at 4°C for 12 h with the appropriate primary antibody (mouse anti-Flag (Sigma), rabbit anti-myc (Sigma), mouse anti-HA (Abmart, China), rabbit anti-GAPDH (Abmart), mouse anti-IRF3 (CST, China), or mouse anti-spho-IRF3 (CST)). Membranes were washed twice with TBST and incubated with HRP-labeled goat anti-rabbit or goat anti-mouse secondary antibody (Abmart) at room temperature for 1 h, and then visualized with Tanon 5200 chemiluminescence imaging system.

### Mammalian two-hybrid assay

Protein interactions were detected by mammalian two-hybrid system as described previously [22]. pFN10A(ACT) and pFN11A(BIND) were purchased from Promega. pFN10A(ACT)-human-IRF3 and pFN11A(BIND)-APPV-N^pro^ were transfected into HEK293T cells using Lipofectamine 3000. The empty vector pBIND was served as controls. Cells were incubated for 24 h at 37°C in the presence of 5% CO_2_, and then the luciferase activity was detected as described above.

### Immunofluorescence assay

HEK293T cells were, respectively, transfected with pCAGGS-Flag-IRF3 and pCAGGS-HA-APPV-N^pro^, or co-transfected with these plasmids for 24 h. The cells were fixed with 4% paraformaldehyde for 15 min, permeabilized with 0.1% Triton X-100 and blocked with 0.5% BSA in PBS. Rabbit anti-Flag and rabbit anti-HA antibodies were used to stain transfected cells, and then incubated with Alexa Fluor 488-conjugated goat anti-rabbit or 568-conjugated goat anti-rabbit secondary antibody. The nuclei were stained with DAPI (Sigma). The images were acquired with fluorescent microscope (Leica, Germany).

### RNA isolation and RT-qPCR

HEK293T cells were transfected with pCAGGS-Flag-APPV-N^pro^ or pCAGGS-Flag-CSFV-N^pro^ for 24 h, followed by stimulation with Poly (I:C) for 12 h. Total RNA was extracted using total RNA Kit I (Takara, China) following the manufacturer’s protocol. The mRNA level of IFN-β was detected by RT-qPCR with SYBR Green Real-Time PCR Master Mixes (Takara). The β-actin was used as the reference gene. All data were showed as relative fold change by the threshold cycle (∆∆Ct) method.

### Statistical analysis

All data were recorded for three independent experiments and analyzed with GraphPad Prism 5.0 by one-way ANOVA or Student’s *t*-test. The error bars represent standard deviations. *p*-values are indicated using asterisks as *p < 0.05, and **p < 0.01.

## Results

### Sequence alignment of pestivirus N^pro^

**Figure 1.**
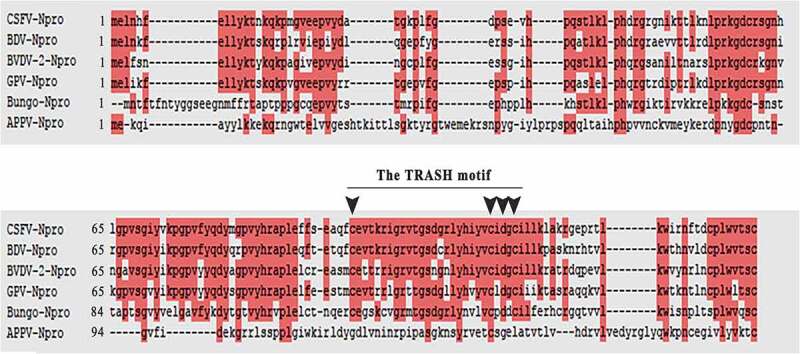
Sequence relationships among different pestiviruses N^pro^. The sequences from GenBank were used to create the sequence alignment: CSFV: KC533775.2; BDV: AHN82189.1; BVDV2: KU756226.1; GPV: NP_620053.1; Bungo: QBC66149.1; and APPV: MH378079. The amino acid residue numbers are indicated on the left of the sequences. The amino acids conserved in all N^pro^ were shown on a red background. The amino acid residues of the functional domain (TRASH motif) in other pestivirus N^pro^ were shown by arrows. The sequences were aligned with ClustalW2, and the figure was prepared with ESPript3.0

The functionality of APPV-N^pro^ compared to other pestivirus N^pro^ is unclear. We evaluated the APPV-N^pro^ sequence and other pestivirus N^pro^ sequences. Representative sequences of pestivirus N^pro^ were aligned using ClustalW2 software (Figure 1). APPV encodes an N^pro^ protein of 21 kDa. The sequence of APPV-N^pro^ is highly variable, but it exhibits some conserved residues. We identified a functional domain (TRASH motif) in other pestivirus N^pro^ that was not present in APPV-N^pro^ [13], indicating that the functional region of APPV-N^pro^ differs from that of other pestivirus N^pro^.

### APPV-N^pro^ antagonized IFN-β production

**Figure 2.**
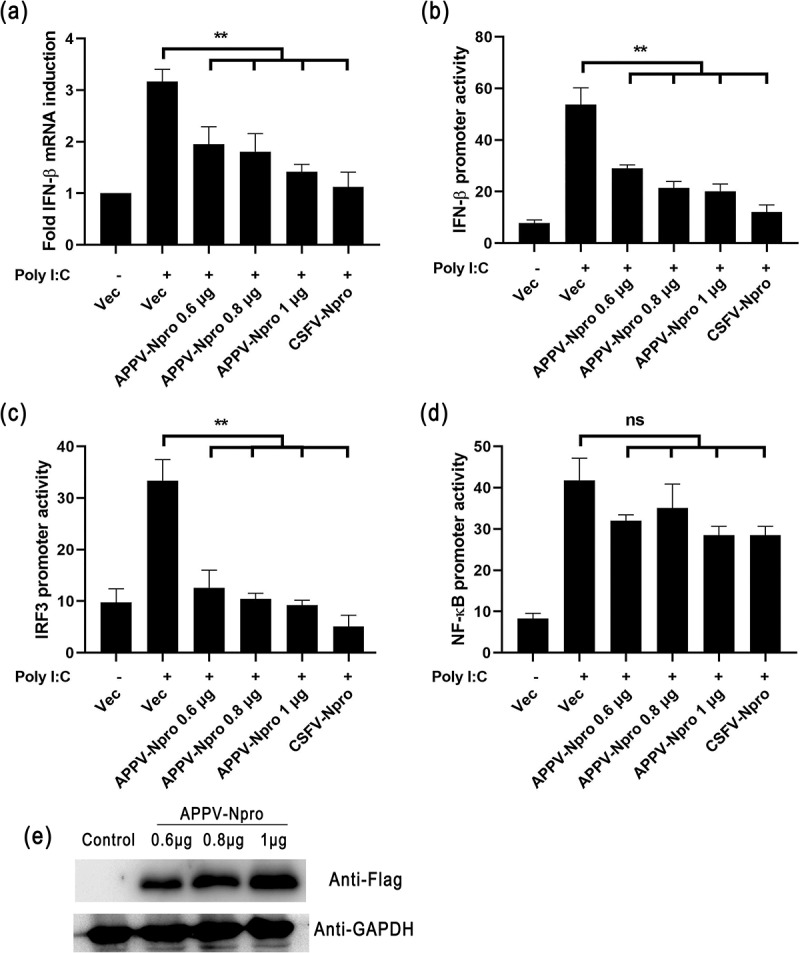
APPV-N^pro^ antagonized the IFN-β production. (a) HEK293T cells were transfected with increasing amounts of pCAGGS-Flag-APPV-N^pro^ or pCAGGS-Flag-CSFV-N^pro^ for 24 h, followed by treatment of Poly (I:C) for 12 h. The mRNA levels of IFN-β were detected by RT-qPCR. (b–d) HEK293T cells were co-transfected with luciferase plasmid (pIFN-β-luc, pIRF3-luc, or pNF-κB-luc), recombinant plasmid (pCAGGS-Flag-APPV-N^pro^ or pCAGGS-Flag-CSFV-N^pro^), and pRL-TK. After 24 h, the cells were treated or mock-treated with Poly (I:C) for 12 h, and then the cell lysates were collected for luciferase assays. Values are presented as the mean ± SD from three independent experiments

To determine the IFN-β production regulated by APPV-N^pro^, HEK293T cells were transfected with increasing amounts of pCAGGS-Flag-APPV-N^pro^ or pCAGGS-Flag-CSFV-N^pro^ (positive control), followed by stimulated with Poly (I:C) for 12 h. The mRNA level of IFN-β and the IFN-β promoter activity were inhibited in APPV-N^pro^-expressed cells compared with the empty vector-transfected cells (Figure 2(a,b)). These results indicated that APPV-N^pro^ contributed to the ability of APPV to inhibit type I IFN production.

### APPV-N^pro^ suppressed IFN-β production by targeting IRF3

The activation of transcription factors IRF3 and NF-κB is essential for IFN-β production [23]. Since the results indicated that IFN-β production could be antagonized by APPV-N^pro^, we studied the effect of APPV-N^pro^ on the IRF3 and NF-κB promoter activity. HEK293T cells were transfected with pCAGGS-Flag-APPV-N^pro^ and the luciferase reporter plasmid IRF3-luc or NF-κB-luc, together with the internal control plasmid pRL-TK. After stimulation with Poly (I:C) for 12 h, there was no antagonism of the NF-κB promoter activity through overexpressing APPV-N^pro^ (Figure 2(d)). In contrast, the IRF3 promoter activity was inhibited by APPV-N^pro^ (Figure 2(c)), suggesting that APPV-N^pro^ might target IRF3 signaling molecules to inhibit the IFN-β signal pathway.

**Figure 3.**
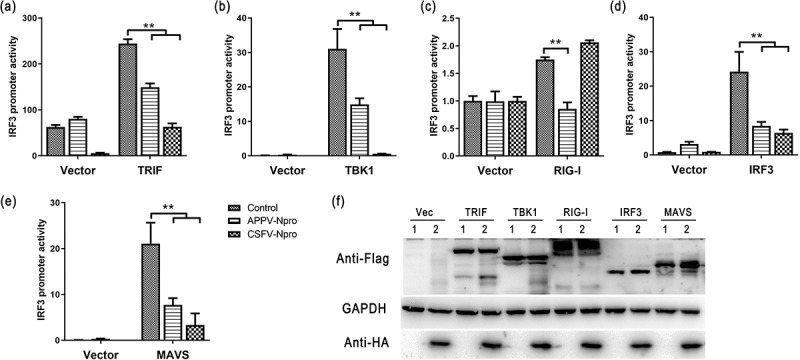
APPV-N^pro^ suppressed IFN-β expression through IRF3. (a–e) The viral protein plasmids (pCAGGS-Flag-APPV-N^pro^ or pCAGGS-Flag-CSFV-N^pro^), innate immunity activators expression plasmids (pCAGGS-Flag-TRIF, pCAGGS-Flag-RIG-I, pCAGGS-Flag-TBK1, pCAGGS-Flag-MAVS, or pCAGGS-Flag-IRF3), together with pIRF3-luc and pRL-TK were co-transfected into HEK293T cells. The IRF3 promoter activity in the transfected cells were detected after 24 h. (f) The expression of proteins was analyzed by western blot in transfected cells. Values are presented as the mean ± SD from three independent experiments

The IRF3 response is initiated by signaling components (such as RIG-I, TRIF, and MAVS). However, the positive adaptor molecule for APPV-N^pro^ is unknown. We constructed human RIG-I, MAVS, TRIF, TBK1, and IRF3 recombinant expression plasmids. These proteins could be detected in HEK293T cells by western blot (Figure 3(f)). We then investigated the potential role of APPV-N^pro^ in the IRF3 promoter activity mediated by these molecules. The luciferase assay results showed that the mediation of IRF3 promoter activity by these molecules was inhibited by APPV-N^pro^ (Figure 3(a–e)). These results indicate that APPV-N^pro^ inhibits IRF3.

### APPV-N^pro^ blocked the activation of IRF3

**Figure 4.**
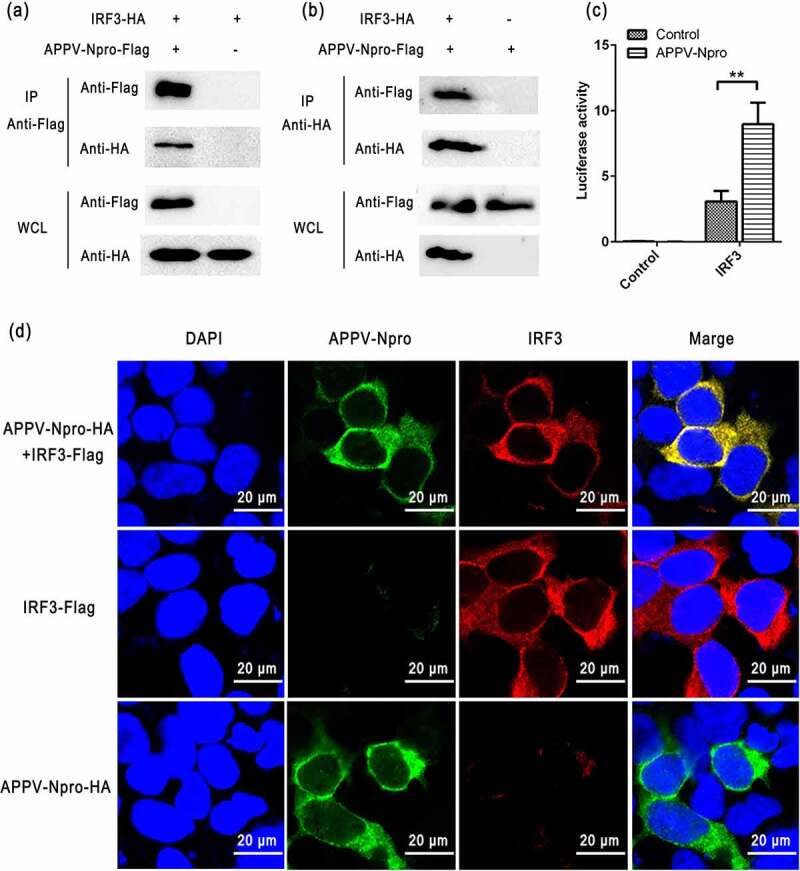
APPV-N^pro^ interacted with IRF3. (a,b) HEK293T cells were co-transfected with pCAGGS-Flag-APPV-N^pro^ and pCAGGS-HA-IRF3. The empty vector pCAGGS-Flag and pCAGGS-HA were used as controls. The immunoprecipitations were collected by anti-Flag M2 magnetic beads or anti-HA magnetic beads and analyzed by western blot using the primary antibodies (mouse anti-Flag and mouse anti-HA antibodies). WCL indicated the analyzation of whole-cell lysate. (c) HEK293T cells were co-transfected with pFN10A(ACT)-IRF3 and pFN11A (BIND)-APPV-N^pro^. The empty vector pBIND served as controls. After 24 h, the luciferase activity of the transfected cells was detected. Values are presented as the mean ± SD from three independent experiments. (d) HEK293T cells were seeded onto coverslips in 24-well plates, and then the cells were transfected with pCAGGS-Flag-APPV-N^pro^ and pCAGGS-HA-IRF3, or co-transfected with them, respectively. After 24 h, the transfected cells were fixed for immunofluorescence assay with APPV-N^pro^ (green), IRF3 (red), and nuclei (blue). Images were obtained using confocal microscopy

To determine if APPV-N^pro^ interferes with IRF3 activation, we studied the interaction of APPV-N^pro^ with IRF3. HEK293T cells were co-transfected with pCAGGS-Flag-APPV-N^pro^ and pCAGGS-HA-IRF3. An indirect immunofluorescence assay demonstrated that Flag-APPV-N^pro^ and HA-IRF3 were co-localized in the cytoplasm (Figure 4(d)). This was followed by Co-IP and western blot using HA and Flag mAbs, and the results showed that HA-IRF3 efficiently co-immunoprecipitated with Flag-APPV-N^pro^ (Figure 4(a,b)). Mammalian two-hybrid assay results confirmed the interaction of APPV-N^pro^ with IRF3 (Figure 4(c)). These results indicated that APPV-N^pro^ interacted with IRF3 in HEK293T cells Figure 4(a).

**Figure 5.**
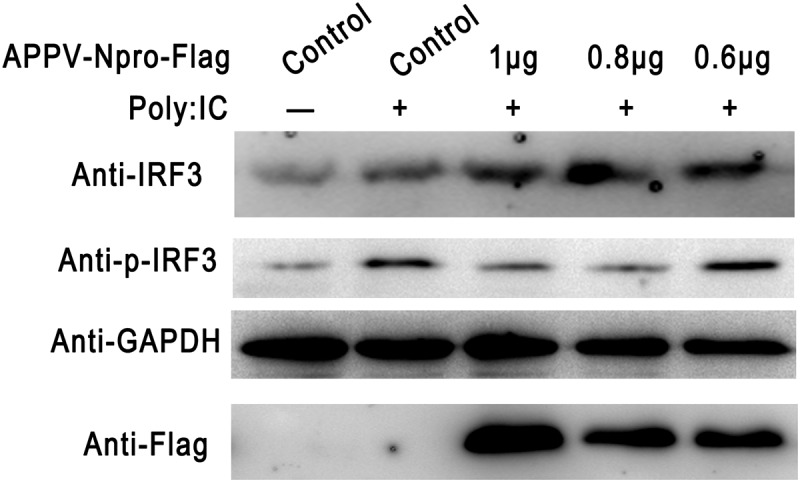
APPV-N^pro^ inhibited the phosphorylation of IRF3. HEK293T cells were transfected with increasing amounts of pCAGGS-Flag-APPV-N^pro^. After 24 h, the cells were treated or mock-treated with Poly (I:C) for 12 h, and then the cell lysates were collected. The expression of IRF3, the phosphorylation of IRF3, GAPDH, and Flag-APPV-N^pro^ were analyzed by western blot

After IRF3 is activated through phosphorylation and nuclear translocation, the coordinated assembly of activated transcription factors and its binding to the promoter of specific defense genes to increase their transcription in the cell nucleus [24]. We also determined the effect of APPV-N^pro^ on the phosphorylation of IRF3 by western blot. Figure 5 shows that the phosphorylated IRF3 was enhanced in Poly (I:C)-treated cells compared with mock-treated cells, but decreased in APPV-N^pro^-expressed cells. These results indicated that APPV-N^pro^ disrupted IFN responses by blocking the activation of IRF3.

### APPV-N^pro^ inhibited the IFN-β production in ST cells

**Figure 6.**
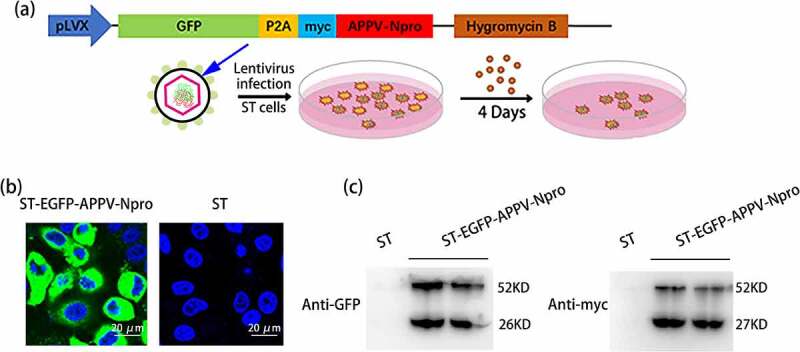
The construction of ST-EGFP-APPV-N^pro^ cell lines. The construction of lentiviral expression plasmid was shown in (a). (b) ST cells were infected by lentiviruses expressing EGFP-APPV-N^pro^, and then subjected to selection with Hygromycin B to generate ST-EGFP-APPV-N^pro^ stable cell line. (c) The ST-EGFP-APPV-N^pro^ cells and ST cells were observed by fluorescence microscope. (d) The expression of fusion protein in the ST-EGFP-APPV-N^pro^ cells were analyzed by western blot using mouse anti-GFP and rabbit anti-myc antibodies

**Figure 7.**
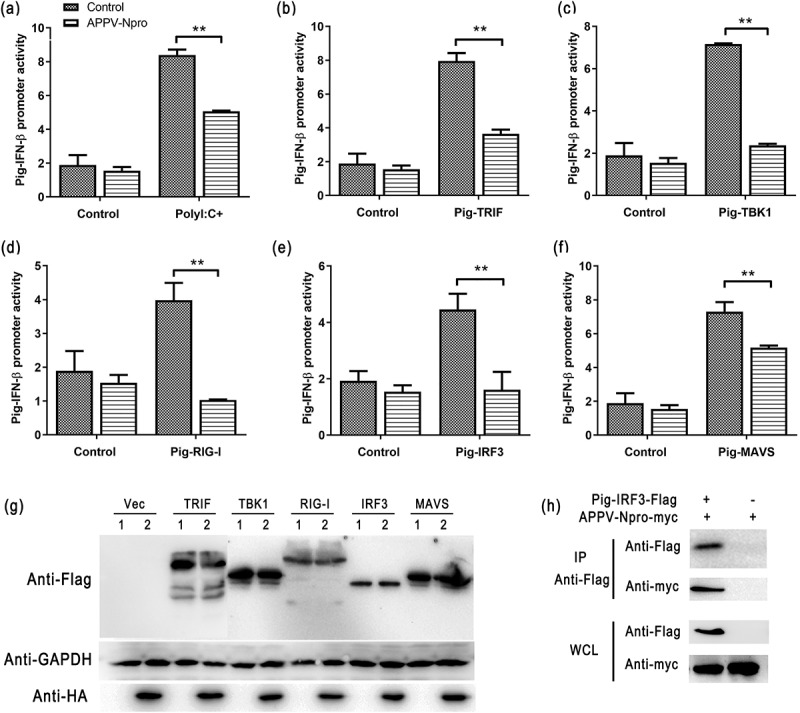
APPV-N^pro^ blocked the activation of IRF3 in ST cells. (a) ST-EGFP-APPV-N^pro^ cells were co-transfected with porcine IFN-β promoter (pPig-IFN-β-luc), pRL-TK, followed by treated or mock-treated with Poly (I:C). Cells were lysed to analyze the promoter activity. (b–g) pPig-IFN-β-luc, pRL-TK, and recombinant plasmids encoding the porcine innate immunity genes were co-transfected into ST-EGFP-APPV-N^pro^ cells, respectively. After 24 h post-transfection, the porcine IFN-β promoter activities were detected by luciferase assays. The expression of proteins was analyzed by western blot in transfected cells. (h) ST-EGFP-APPV-N^pro^ cells were co-transfected with pCAGGS-Flag-Pig-IRF3, the pCAGGS-Flag was used as negative control, and then the immunoprecipitations were collected and analyzed by western blot. The whole-cell lysate was indicated by WCL

The experiments above were all performed on human cells, we investigated whether the production of IFN-β was also suppressed by APPV-N^pro^ in porcine cells. The ST-EGFP-APPV-N^pro^ cell line was constructed using lentivirus (Figure 6(a)). The expression of EGFP-APPV-N^pro^ fusion protein was confirmed by confocal microscopy and western blot (Figure 6(b,c)). Then, we detected the activity of porcine IFN-β promoter in ST-EGFP-APPV-N^pro^ cells [25]. Five recombinant plasmids expressing porcine RIG-I, TRIF, MAVS, TBK1, and IRF3 were constructed. After co-transfection with the recombinant expression plasmid, pPig-IFN-β-luc, and pRL-TK, the porcine IFN-β promoter activity was inhibited by APPV-N^pro^ (Figure 7(a–f)). Protein expression was confirmed by western blot (Figure 7(g)). In addition, pCAGGS-Flag-Pig-IRF3 was transfected into ST-EGFP-APPV-N^pro^ cells, and the cells were collected for Co-IP assays. The results showed that APPV-N^pro^ could specifically interact with porcine IRF3 (Figure 7(h)). Therefore, the APPV-N^pro^ also antagonized the production of IFN-β through IRF3 in porcine cells.

### The essential region of APPV-N^pro^ blocking the IRF3 activation

**Figure 8.**
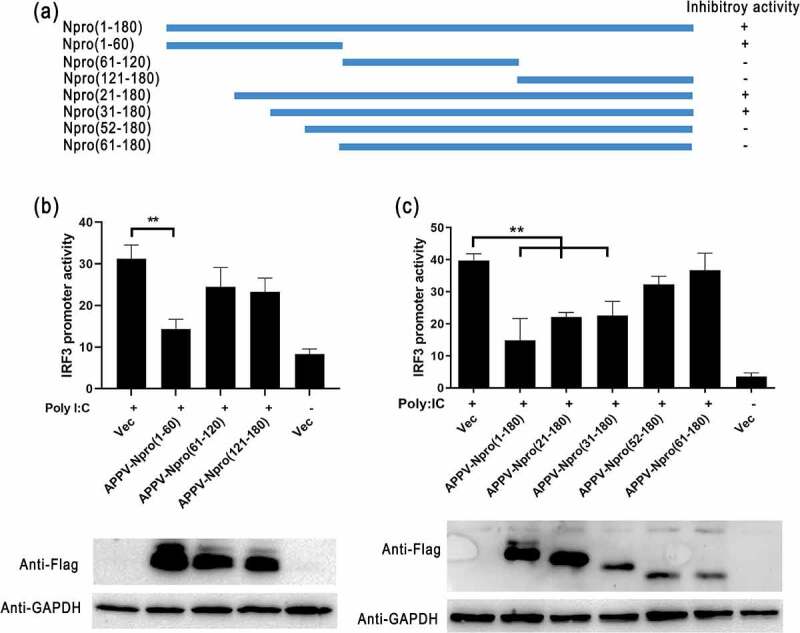
The region of APPV-N^pro^ inhibited the IRF3 promoter activity. (a) The truncated APPV-N^pro^ were amplified from the full-length APPV-N^pro^, and then inserted into the pCAGGS-Flag vector, the inhibitory activity of IRF3 promoter is indicated by + (positive) or – (negtive). (b,c) HEK293T cells were co-transfected with pIRF3-luc, pRL-TK and truncated APPV-N^pro^ plasmids for 24 h, followed by treatment of Poly (I:C) for 12 h, and then the cells were lysed to analyze the promoter activity. Values are presented as the mean ± SD from three independent experiments. The expression of truncated APPV-N^pro^ in cell lysates were detected by western blot using rabbit anti-Flag antibody. GAPDH was used as a loading control

To identify the key region in APPV-N^pro^ that was responsible for antagonizing the IFN-β production, a panel of truncated APPV-N^pro^ plasmids were constructed (Figure 8(a)). We then analyzed the IRF3 promoter activity by transfecting with truncated APPV-N^pro^ plasmids, pIRF3-luc, and pRL-TK in HEK293T. Unexpectedly, only the N-terminal 60 aa of APPV-N^pro^ suppressed the IRF3 promoter activation as the full-length APPV-N^pro^ (Figure 8(b)). For further investigation, another panel of truncated APPV-N^pro^ recombined plasmids were constructed (Figure 8(a)). Luciferase assays results showed that both APPV-N^pro^ (21–180 aa) and APPV-N^pro^ (31–180 aa) truncation inhibited IRF3 promoter activity. In contrast, APPV-N^pro^ (52–180 aa) and APPV-N^pro^ (61–180 aa) did not block IFN-β induction (Figure 8(c)).

**Figure 9.**
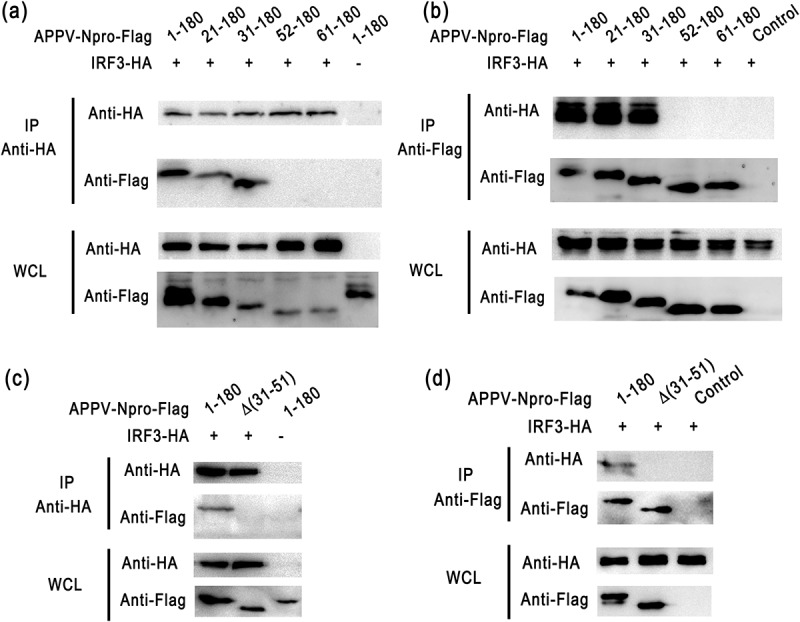
The truncated APPV-N^pro^ interacted with IRF3 protein. (a,b) HEK293T cells were co-transfected with pCAGGS-HA-IRF3 and truncated APPV-N^pro^ recombinant plasmids pCAGGS-Flag-APPV-N^pro^(1–180aa), pCAGGS-Flag-APPV-N^pro^(21–180aa), pCAGGS-Flag-APPV-N^pro^(31–180aa), pCAGGS-Flag-APPV-N^pro^(52–180aa), or pCAGGS-Flag-APPV-N^pro^(61–180aa), the empty vector pCAGGS-Flag was used as control. After 24 h, the cells were collected and co-immunoprecipitations were performed with anti-Flag M2 magnetic beads or anti-HA magnetic beads. The expression of proteins in the immunoprecipitations were analyzed by western blot. WCL indicated the whole cell lysate was analyzed. (c,d) The APPV-N^pro^ variant with deletion of amino acids 31–51aa from the N-terminus were constructed. The pCAGGS-HA-IRF3 and truncated APPV-N^pro^ recombinant plasmids pCAGGS-Flag-APPV-N^pro^(1–180aa), pCAGGS-Flag-APPV-N^pro^(∆31-51aa) were co-transfected into HEK293T cells. Co-immunoprecipitations were performed and the expression of proteins in the immunoprecipitations were analyzed by western blot. The whole-cell lysate was indicated by WCL

To detect whether the N-terminal of APPV-N^pro^ was the IRF3 binding domain, we co-transfected IRF3 and full-length or truncated APPV-N^pro^ recombined plasmids into HEK293T cells for 24 h, and then treated with Poly (I:C). The lysates from transfected cells were collected to detect the interaction between truncated APPV-N^pro^ and IRF3. IRF3 efficiently co-immunoprecipitated with APPV-N^pro^ (1–180 aa), APPV-N^pro^ (21–180 aa), and APPV-N^pro^ (31–180 aa), but not with other truncated APPV-N^pro^ (Figure 9(a,b)). To confirm this result, an APPV-N^pro^ mutation with deletion of amino acids 31–51 aa from the N-terminus was constructed. After co-transfection with the IRF3-recombined plasmid, we found that removal of 31–51 aa from the N-terminus of APPV-N^pro^ eliminated its ability to interact with IRF3 (Figure 9(c,d)). These results indicated that APPV-N^pro^ interacted with IRF3 through its N-terminal residues (31–51 aa).

## Discussion

APPV was a new porcine pestivirus discovered recently, and the number of pigs positive for APPV was high depending on epidemiological surveys [26,27]. Pestiviruses can cross the placenta, infect the fetus, and result in the birth defect of infected animals [14–16]. APPV is linked to cases of CT type A-II in newborn piglets, but its pathogenesis is not fully known. In addition, APPV is often found with other viruses in persistently infected animals, and it may intensify these secondary infections. Piglets showing CT had a high APPV viral load from birth to six months of age in tests using specific antibodies to APPV NS3 [17]. Cagatay et al. monitored CT unaffected and affected piglets from birth to slaughter and found that APPV could be maintained in pig herds and transmitted horizontally and vertically [28]. Hence, APPV infection may lead to virus persistent infection, partially owing to its ability to circumvent host innate immunity.

The APPV strains are distinct from the other pestiviruses in their genomic sequence [17,29,30]. Phylogenetic analysis showed a low similarity (25%–28%) to well-known pestiviruses including CSFV, BVDV, and BDV. The N^pro^ of BVDV and CSFV appear to be involved in regulating host immune responses. As demonstrated in the sequence alignment of pestiviruses N^pro^, APPV-N^pro^ was also distinct from the other pestiviruses, and there were only a few conserved residues. It is unknown whether APPV-N^pro^ plays an important role in virus innate immune evasion. We found that the mRNA level and promoter activity of IFN-β were both inhibited in APPV-N^pro^-expressed cells. APPV-N^pro^ targeted IRF3 to block IFN-β induction. These results were consistent with those of Gil et al. who found that N^pro^ overexpression down-regulated IFN induction [20–22].

Many viruses using its encoded proteins to target IRF3 and counteract IRF3 functions. These viral proteins include the leader protein of Theiler’s murine encephalomyelitis virus, the N^pro^ protein of pestiviruse, the NS1 and NS2 proteins of bovine respiratory syncytial virus, and the ML protein of Thogoto virus [31]. These viral proteins employ various mechanisms for IRF3 antagonism, such as inhibiting the phosphorylation of IRF3, disturbing the translocation of IRF3 from cytoplasm into the nucleus, and targeting IRF3 for proteasomal degradation [31]. CSFV and BVDV are closely related viruses, and their N^pro^ are 70% identical. However, CSFV and BVDV-N^pro^ employ different mechanisms of the IFN evasion. CSFV-N^pro^ is able to down-regulate the IFN induction through blocking the transcription of IRF3, as well as inducing ubiquitination and proteasome-dependent degradation of IRF3 [13,22,32,33]. BVDV-N^pro^ does not appear to disturb the transcription of IRF3 gene, but it mainly inhibits the DNA binding activity of IRF3 and induces IRF3 degradation [22,32]. In the present study, APPV-N^pro^ blocked IRF3 transcription by interacting with IRF3, but it was unable to induce IRF3 degradation (unpublished data).

The pestiviruses N^pro^ targeting IRF3 usually depended on a TRASH domain (Cys-X21-Cys-X3-Cys) in the C-terminal half of N^pro^ [13,22,33]. The zinc-binding TRASH domain of CSFV is indispensable for binding IRF3, which could bind to a cellular protein induce the ubiquitination reaction and the proteasomal degradation of IRF3. Other studies have reported that amino acid residues at the N-terminal N^pro^ were also responsible for the IFN-β antagonism in pestiviruses [14,19,31]. Amino acid residues at the positions 40 H, 17P, and 61 K of CSFV-N^pro^, and 8 L, 22E, and 49 H of BVDV were important for their IFN-β antagonism. However, there was no discernable TRASH domain in APPV-N^pro^ as described in the other pestivirus N^pro^ [2]. This might explain why no degradation of IRF3 was detected in APPV-N^pro^-expressed cells. These data indicated that the functional region of APPV-N^pro^ might differ from other pestiviruses. We constructed a series of truncated APPV-N^pro^ recombinant plasmids, and the IRF3 promoter activities in truncated APPV-N^pro^-expressed cells were studied. Removal of the N-terminal 31–51 aa eliminated its ability to interact with IRF3. The IRF3 promoter activity was not inhibited in the APPV-N^pro^ (31–51 aa)-expressed cells. This suggested that N-terminal 31–51 aa of APPV-N^pro^ was a key region, but not the independently functional region, because it still required the participation of other amino acids. Furthermore, the unclassical domain in APPV-N^pro^ might explain why APPV-N^pro^ was unable to induce the same inhibition of the transcript level and the promoter activity of IFN-β as CFSV-N^pro^. Thus, the main mechanism of APPV-N^pro^ suppressing the IFN-β response was different from other pestiviruses.

In summary, we investigated the effect of APPV-N^pro^ on regulating the production of IFN-β. APPV-N^pro^ reduced IFN-β production mainly through blocking IRF3 activation. The N-terminal 31–51 aa of APPV-N^pro^ was involved in the counteraction of IFN-β induction. This study revealed the function of APPV-N^pro^ in inhibiting the innate immune response, which may be responsible for the persistent infection of APPV.
